# The oesophageal diverticulum of *Dirioxa pornia* studied through micro-CT scan, dissection and SEM studies

**DOI:** 10.1186/s12896-019-0585-8

**Published:** 2019-12-18

**Authors:** Kala Bhandari, Peter Crisp, Michael A. Keller

**Affiliations:** 1 School of Agriculture, Food and Wine, The University of Adelaide, Adelaide, SA Australia; 2 Entomology Unit, South Australian Research and Development Institute, PIRSA, Adelaide, SA Australia

**Keywords:** Island fly, Tephritidae, 3D micro-imaging, Avizo® fire, Bacteria

## Abstract

**Background:**

*Dirioxa pornia* (Diptera, Tephritidae) (Island fly) is an Australian native species related to a number of pestiferous fruit flies but, unlike many of the pest species, has not been studied extensively due to its non-pest status. However, due to *D. pornia*’s apparent reliance on the bacteria for survival it is an ideal species to undertake studies into interaction between Tephritid species and bacteria associated with the intestinal tract. The oesophageal diverticulum, which is a blind-ended protrusion of the oesophagus, has been studied, described and characterised in many other Tephritid species. Unlike many other species where the oesophageal diverticulum has been observed the organ was only observed in male *D. pornia*. It is speculated that this sexual dimorphism the organ may be the primary location to host beneficial bacteria in the involved in the production of the nuptial gift and the mating success of this Tephritid species. In case of *D. pornia*, however, no study on any area of the digestive system has been conducted. This study was conducted to locate and characterize the oesophageal diverticulum in *D. pornia*. A virtual dissection of the alimentary tract was made through micro-computer tomography studies. These studies were followed by dissection and scanning microscopy studies to elucidate the presence of bacteria.

**Results:**

The oesophageal diverticulum of *D. pornia* is part of the foregut and distends from the oesophagus within the head of the fly. The shape of the oesophageal diverticulum corresponds with the *Ceratitis* type. Scanning microscopy studies of the oesophageal diverticulum show rod-shaped bacterial cells residing along with yeast cells in the lumen. The organ was only observed in male specimens.

**Conclusions:**

This study classifies the oesophageal diverticulum of *D. pornia* under the “*Ceratitis type*” of oesophageal diverticula in Tephritid species. The study also proves that micro-CT scanning is possible to locate soft tissues in Tephritid species and the Avizo® Fire software can be successfully used to visualize 3 dimensional (3D) images from x-rays. The methods used in this experiment can be used in future studies for visualising soft tissues of adult Tephritid species through micro tomography. There is sexual dimorphism with the organ only found in males. Finally this study shows that bacteria are present in the oesophageal diverticulum of *D. pornia*.

## Background

Fruit flies (Diptera; Tephritidae) constitute some of the major pests of horticultural crops. Control measures such as the sterile insect technique (SIT) are used to keep populations of many fruit fly species within manageable limits. In Australia SIT is used both as management and eradication tool for the *Bactrocera tryoni* (Froggatt) (Queensland fly) and *Ceratitis capitata* (Wiedemann) (Mediterranean fly). To improve the efficacy of a new facility for the production of *B. tryoni*, for use in SIT programs, new rearing methods and diets are being developed. There are various published studies dedicated to the enhancement of SIT practices, including the potential of the exploitation of gut bacteria to improve on rearing efficacy and fly fitness [1–3].

Insects have been shown to have a range of complex relationships with bacteria varying from casual interactions to complete dependence [4, 5]. Non-pathogenic mutualistic relationships between insects and their gut bacteria are reported to mainly involve nutritional interactions [6–8]. In some cases the relationship is so critical that when the bacteria are eliminated from their host, the host cannot survive on its natural food source alone [9].

*Bactrocera oleae* (Rossi) (Olive fly) is the first example of a Tephritid known to host symbiotic bacteria. Petri, in as early as 1909, hypothesised that the *B. oleae* gut-symbionts multiply in the oesophageal diverticulum (oesophageal bulb/ pharyngeal vessel) and are released into the gut to be digested by the fly [10]. The oesophageal diverticulum was considered exclusive to the Olive fly until 1973, when Girolami discovered and described the organ in most Tephritid species [11], as follows,

- The ‘*Dacus*’ type. The *Dacus* type is the largest of the oesophageal diverticula and the bacteria multiply near the elongated basal epithelial cells.
- The ‘*Ceratitis*’ type. This type is present in Trypetinae and Dacinae, except *B. oleae*. The bacteria, which are easily visible in Trypetinae, multiply in the lumen.
- The ‘*Ensina*’ type. This is egg-shaped and present in Tephritinae, except Terellini. Bacteria are not known to be present in this oesophageal diverticulum.
- The ‘*Chaetorellia*’ type. This is present in the tribe Terellini and the features combine some of the characteristics of the *Ceratitis* and *Ensina* types.

The oesophageal diverticulum has also been studied in the Apple maggot, *Rhagoletis pomonella* (Walsh) [12, 13]. The studies hypothesise that the oesophageal diverticulum is present in order to house symbiotic bacteria which are released into the gut lumen as and when required by the host insect. This hypothesis is similar to those made by Petri and Girolami [10, 11].

*Dirioxa pornia* (Walker) (Tephritidae: Phytalmiinae) is a fruit fly native to Australia and New Caledonia that lays its eggs in damaged fruit [14]. However, a small number of cases of *D. pornia* larvae being found in exported citrus have been reported from overseas markets which lead to initial laboratory studies in South Australia. In countries such as Thailand and New Zealand it is listed as a quarantine pest. It was found that unlike other fruit fly species (such as *C. capitata* and *B. tryoni*), *D. pornia* cultures could only be maintained when their diets were supplemented with artificially grown gut-bacteria [15]. There have been only limited studies on this species [16, 17]. The study on the structure of the alimentary tract, including the oesophageal diverticulum has not been made. Elucidating the relationship between the oesophageal diverticulum and bacteria in this species may provide a better understanding of the importance of bacteria in the biology of Tephritid species in general, which in turn could be used in improving efficiency in SIT mass rearing facilities.

Earlier in this study the precise location of the oesophageal diverticulum could not be established through dissection methods alone. To accurately elucidate the location of the oesophageal diverticulum micro-computed tomography (micro-CT) scan of the head of adult *D. pornia* was conducted. Micro-CT is an emerging technology for the imagery of insects [18, 19]. An advantage that the micro-CT technique has over classical dissection methods is that it is non-destructive and is precise in terms of organ location. Additionally, a single scan can be saved digitally instead of having to physically preserve samples in chemical substances that may either be toxic or can deteriorate over time. Mainly, this technique was selected in order to develop methods to conduct micro-CT scans on Tephritid species.

This study will provide a clear understanding of the structure and location of the oesophageal diverticulum of *D. pornia* and contribute to the development of micro-computed tomography as a non-destructive dissection method to study the structure within Tephritidae and other insects. It also aims to elucidate the presence of bacterial cells within the oesophageal diverticulum of the *D. pornia* through scanning electron micrographs. This information will contribute to the understanding of the relationship between Tephritid fruit flies and the communities of bacteria in their gut and provide a basis for ongoing research in the field.

## Methods

### Island fly culture

Island flies used for the scans were obtained from cultures maintained at the Waite Insectary, School of Agriculture Food and Wine, University of Adelaide, Urrbrae, South Australia, at 27 °C; RH 70–80% and L:D 13:11 (natural light supplemented by fluorescent lighting). Flies for the culture were sourced from Waikerie and Mypolonga in South Australia’s Riverland, male and female flies were trapped in McPhail traps containing Putrescine (FFP) and ammonium acetate (FFA) lures (Suterra LLC, Bend, OR, USA). Adult flies were provided with a diet of hydrolysed yeast and water ad libitum supplemented with *Enterobacter spp* isolated from the gut of wild *D. pornia* collected from Loxton in 2011 [15]. Fresh oranges were poked with the help of a needle and placed in adult cages for oviposition and larval development.

### Preliminary dissections

Prior to CT and SEM scans a series of 100 dissections, 50 male and 50 female, of adult *D. pornia* were conducted with the help of a Nikon SMZ25 stereomicroscope. The age of male and females flies dissected in this manner ranged from 24 h to 3 weeks post eclosion to observe any developmental changes that may have affected outcomes.

### Preparation of island flies for CT scans

Methods for dehydration of the flies were modified from Alba-Trecedor [20]. Our method differs from his mainly in the fixing and dehydration process. In our case, when the samples were fixed with 4% Paraformaldehyde in PBS + 4% Sucrose at a pH of 7.2 prior to dehydration as described in his method, the resulting images appeared unclear. This may have occurred due to fluids trapped during the fixing process. Therefore we did not fix the sample. Instead we directly dehydrated the sample in a graded series of ethanol. Dehydrating of the sample at 70% which was the method used by Alba-Trecedor also resulted unclear images and tissue damage. In order to avoid this we dehydrated the specimen using a graded series of ethanol at 30, 50, 70, 80, 90 and 100% which showed better results. Similarly, staining the sample with 1% Iodine for more than 6 h showed better contrast of the softer tissues in the CT scans than staining them with 1% Iodine for 3 h only.

For this study, 5 day-old male flies were selected from the culture and euthanized at − 18 °C for 3 min. Euthanized flies were dehydrated in a graded series of ethanol starting at 30, 50, 70, 80, 90 and 100% for 30 min each. The dehydrated flies were then stained with 1% iodine in 100% ethanol for over 6 h. The stained flies were then critically dehydrated in hexamethyldisilazane (HMDS) for 2 h with one fresh change of HMDS in between. They were left to dry overnight under a fume hood. One fly per scan was selected and then mounted over the top of the axis of a micro-CT scanner (SkyScan 1072, Bruker microCT, Belgium) by sticking it with Araldite® glue (Selleys®_,_ Padstow, New South Wales, Australia) and left to dry for 20–30 min.

### Micro-CT scanning in SkyScan 1072

The micro-CT scan and analysis of the head of *D. pornia* was performed at Adelaide Microscopy (Medical School North, Frome Road, The University of Adelaide, SA, Australia) with a Skycan 1072 (Bruker microCT, Kontich, Belgium). The specifications used were; beam energy set at 23 kV, current set at 120 μA, cross-section pixel size set to of 3.67 μ; exposure set at 3.4 s and rotation set to 180° with images captured every 0.225°.

### Post-scan image processing

Scanned images that were saved in tagged image file format (TIFF) were reconstructed with the help of the NRecon software (Bruker microCT, Kontich, Belgium). The region of interest (ROI) was identified and the dynamic range selected, following which the image was adjusted for misalignment compensation and in this instance fine tuning/ beam hardening was performed. The image data were then converted into bitmap (BMP) format. The new reconstructed image data were then used to segment, recolour and visualise the alimentary tract of the fly in 3D with the help of the Avizo® Fire 8.1 software (ThermoFisher Scientific Hillsboro, Oregon, USA). A volume rendering module was attached to the data file and the alpha scale adjusted in order to reveal a semi-transparent scanned image of the fly. This was followed by the creation of a label field. An interactive segmentation was performed for the various structures and organs of the scanned 3D image. After the segmentation was complete the new surface generated was visualised with the help of the surface-view and volume rendering modules. Images were captured and saves as TIFF files.

### Dissection of *D. pornia* to study the alimentary tract

Seven 24 h-old adult males were removed from the culture and provided with dyed sucrose solution (Queen Rainbow Food Colours, Australia) for 12 h. One adult was selected and dissected with the help of a Nikon SMZ25 stereomicroscope. The dye helped locate the alimentary tract during dissection. Images of the dissected areas were captured with the help of the attached camera.

### Scanning electron microscopy studies of the oesophageal diverticulum of *D. pornia*

For the SEM studies, 8 adult male *D. pornia* collected from an orchard at Waikerie, SA, were selected. The flies were caught on the leaves and immediately chilled to transportation and processing. The oesophageal diverticuli were removed and fixed in in 4% Paraformaldehyde in PBS, 4% sucrose at a pH of 7.2 for 30 min. 2–3 washes were made in PBS + 4% sucrose for 5 min each. The washed oesophageal diverticuli were then post-fixed in 2% OsO_4_ in PBS for 1 h. They were then dehydrated in a graded series of ethanol at 70, 90 and 100% with 2 changes per concentration of ethanol at a 15 min interval, with an additional third change made for the last concentration of 100% ethanol. This was followed by critical point dehydration in a Bal-Tec CPD 030 Critical Point Dryer. The dried oesophageal diverticuli were then mounted on SEM stubs and coated with platinum. They were observed under a Philips XL20 Scanning electron microscope set at beam energy of 10 keV the next day. Images were captured with the help of the CCD camera and were saved in TIFF format.

## Results

### Preliminary dissections

During preliminary dissections made on the *D. pornia* adults, it was noted that no female flies of the 50 dissected had the oesophageal diverticulum, but 48 of the 50 males dissected were seen to have them. The absence of detection of the diverticulum on 2 males may have been the result of the dissection technique or other handling errors. The age of male and females flies dissected in this manner ranged from 24 h to 3 weeks post eclosion, the 2 males were the Oesophageal Diverticulum was not detected were not of a single age cohort. This finding has led to another study on the sexual dimorphism in *D. pornia*, which will be published in future. Further as part of the scanning technique optimisation 5 male and 2 female flies were prepared, scanned and the images at least partially processed, results presented here are from one male but were typical of all males studied, no diverticulum was detected in either female scanned.

### Micro-CT images

**Fig. 1:**
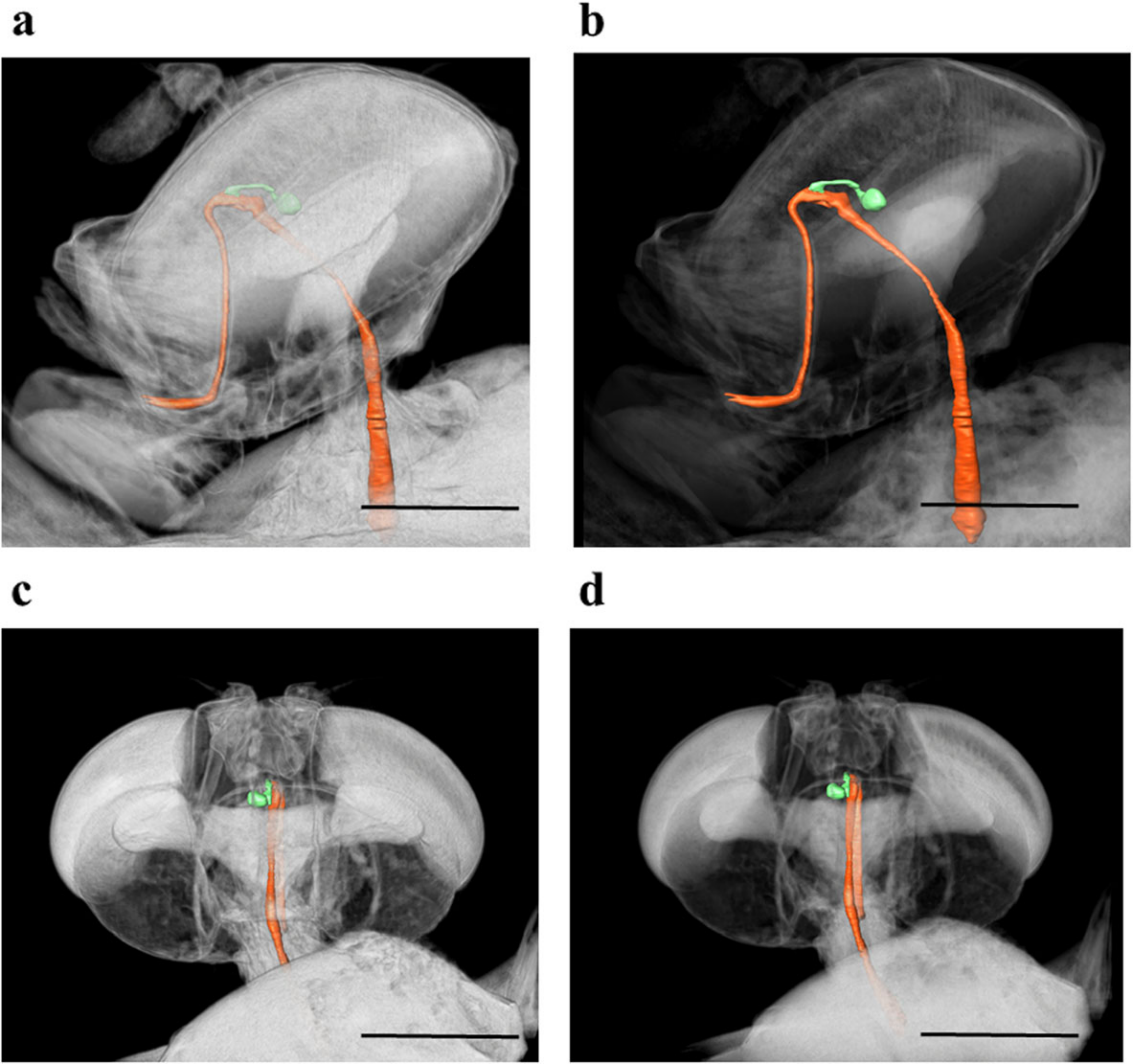
A three dimensional reconstruction of the section of the alimentary tract within the cephalic region of *Dirioxa pornia*. Lateral view (**a**, **b**); dorsal view (**c**, **d**). The oesophageal diverticulum is highlighted in green and the rest of the alimentary tract in orange. The alpha scale manipulated between **a** (0.0279) & **b** (0.0079) and **c** (0.0489) & **d** (0.0189) using the Avizo® Fire 8.1 software edition to compare the location of the oesophageal diverticulum against surrounding tissues and organs. μCT Scan performed in SkyScan 1072 set at 24 keV, 120 μA, image resolution of 3.4 μ; images captured at 180° rotation of 0.225° steps each. Scale bar = 0.5 mm

**Fig. 2:**
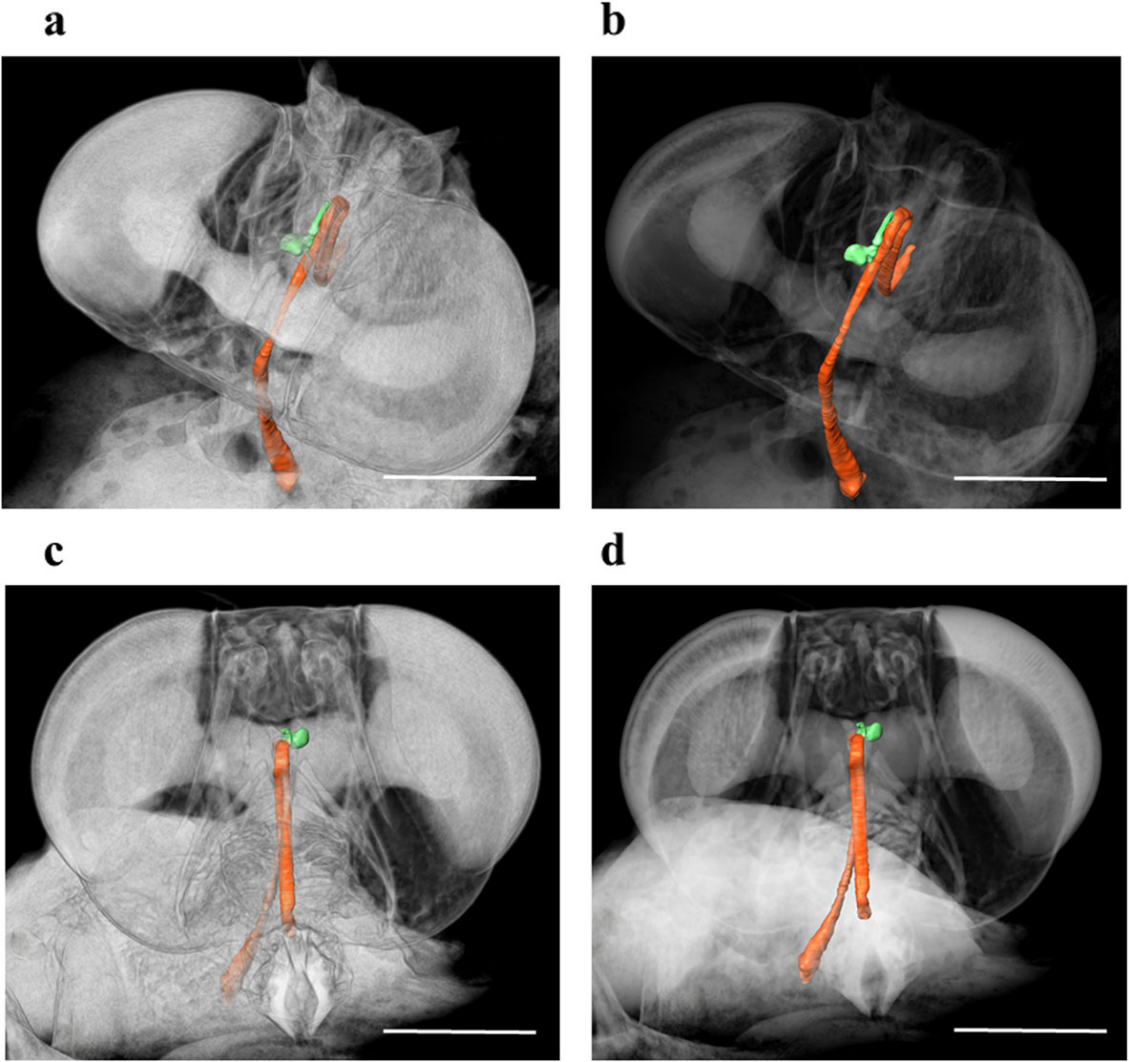
A three dimensional reconstruction of the section of the alimentary tract within the cephalic region of *Dirioxa pornia*. Posterior-superior view (**a**, **b**); anterior view (**c**, **d**). The oesophageal diverticulum is highlighted in green and the rest of the alimentary tract in orange. The alpha scale has been manipulated between **a** (0.0339) & **b** (0.0089), and **c** (0.0339) & **d** (0.0139) using the Avizo® Fire 8.1 software edition to compare the location of the oesophageal diverticulum against surrounding tissues and organs. μCT Scan performed in SkyScan 1072 set at 24 keV, 120 μA, image resolution of 3.4 μ; images captured at 180° rotation of 0.225° steps each. Scale bar = 0.5 mm

Successful 3D micrographs of the oesophageal diverticulum of *D. pornia* were developed with the help of the Avizo® Fire 8.1 software. The oesophageal diverticulum was coloured green and the other regions of the alimentary tract coloured orange. To visualize the position of the oesophageal diverticulum in relation to other organs, the alpha scale (a toggle feature found in the *Volume Rendering* module under *Project View* of the software application) was manipulated when the image was viewed from different angles. This helped in marking the precise location of the oesophageal diverticulum. The oesophageal diverticulum is located in the anterior area of the head of the adult *D. pornia*. It is positioned proximal to the left hemisphere of the brain by branching out from the oesophagus (Figs. 1 & 2).

### Dissection of the alimentary tract of *D. pornia*

**Fig. 3:**
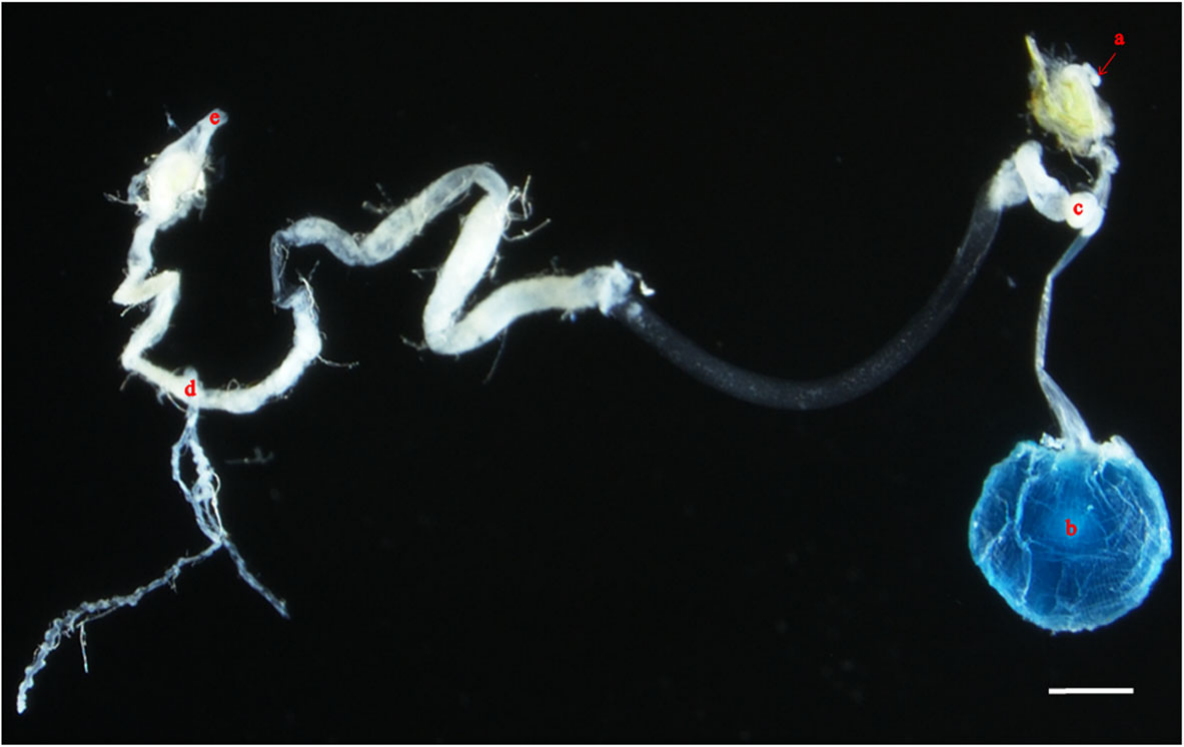
A photographic image of the dissected alimentary tract of *D. pornia* (viewed with a Nikon SMZ25 microscope). The crop is filled with blue dyed sugar solution fed to the fly prior to dissection (for contrast). **a**; oesophageal diverticulum, **b**; crop, **c**; cardia, **d**; Malpighian tubule, **e**; rectum. Stomodaeum (**a**-**c**), mesenteron (**c**-**d**), proctodeum (**d**-**e**). Scale bar = 0.5 mm

With the help of the new found location of the oesophageal diverticulum, it was possible to successfully dissect the entire alimentary tract including the oesophageal diverticulum from the cephalic area. The stomodaeum or the foregut consists of the labella, pharynx, oesophagus, oesophageal diverticulum, crop and cardia. The mesenteron extends from the cardia up to the Malpighian tubules, and the proctodeum continues from the pylorus and Malpighian tubules through the ilium, colon and up to the rectum (Fig. 3).

### SEM studies of the oesophageal diverticulum

**Fig. 4:**
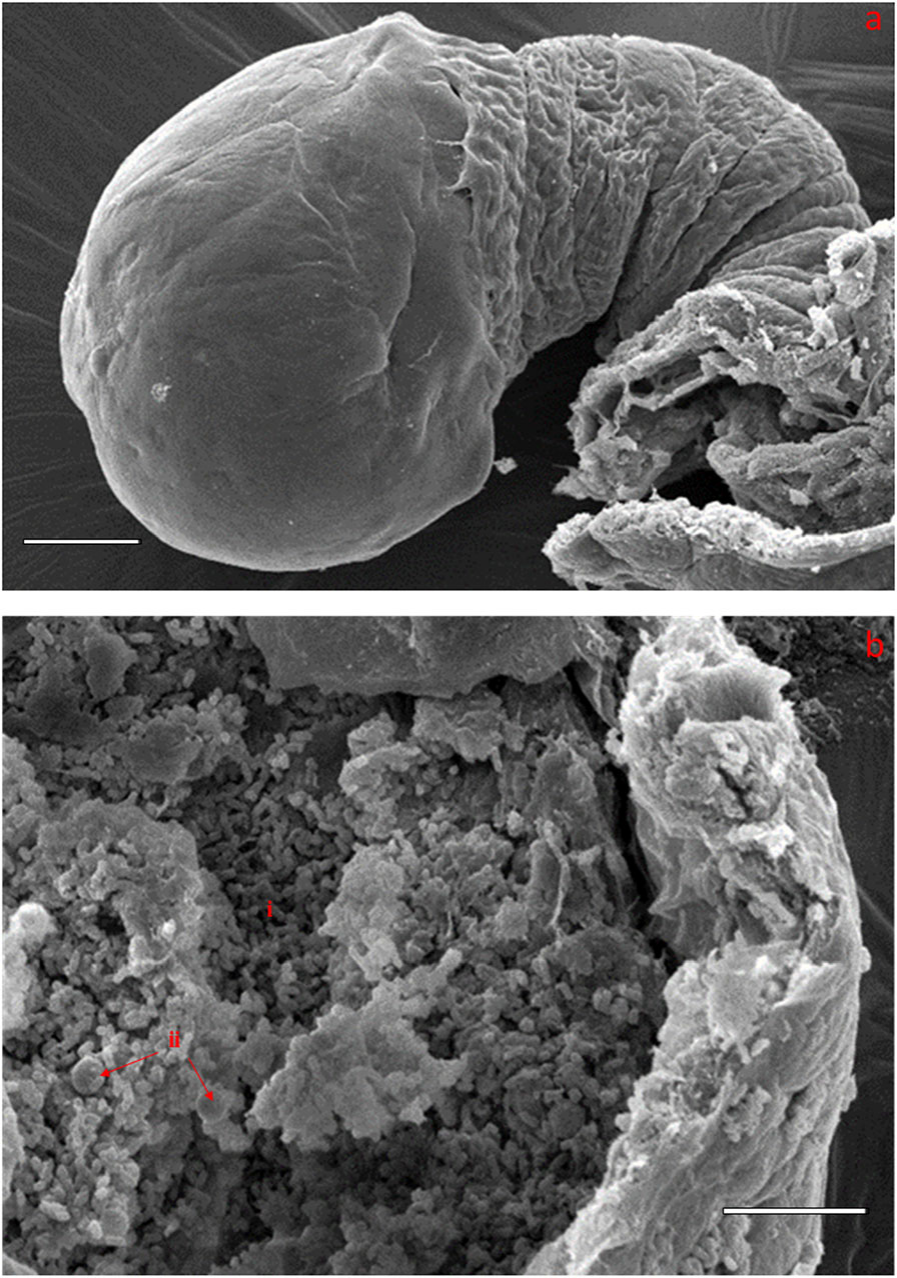
**a**. A SEM micrograph of the oesophageal diverticulum of *D. pornia*. The shape corresponds to the ‘*Ceratitis*’ type. Scale bar = 20 μ. **b**. An opened oesophageal diverticulum of *D. pornia* showing rod-shaped bacterial mass filling the lumen (i). Yeast cells (ii) are also visible. SEM used, Philips XL20, Beam energy set at 10 keV. Scale bar = 10 μ

SEM images of the oesophageal diverticulum reveal a clear visual of the bulb-shaped oesophageal diverticulum (Fig. 4a.). Slicing the bulb with the help of a scalpel blade reveals rod-shaped bacterial mass filling the lumen. Isolated yeast cells are also visible alongside the bacterial cells (Fig. 4b).

## Discussion

In our studies, the dissection of the complete alimentary tract (including the oesophageal diverticulum) of the adult *D. pornia,* were more precise after obtaining results of the micro-CT scan. This is because of the newfound knowledge of the precise location of the oesophageal diverticulum, which helped to be extra careful during dissections. The tissues connecting the foregut to the oesophageal diverticulum can be easily torn-apart because of which the oesophageal diverticulum was not found in many previous dissections. The alimentary tract of the adult male *D. pornia* does not have any remarkable difference from that of *C. capitata* [21]. The scanning electron micrographs of the oesophageal diverticulum of *D. pornia* reveal that the oesophageal diverticulum corresponds to the *Ceratitis* type under the classification system of oesophageal diverticula [11]. Besides the shape, the bacterial mass that fills the lumen of the oesophageal diverticula also correspond with that of *C. capitata* [21].

The presence of uniform rod shaped bacteria inside the lumen of the oesophageal diverticula vaguely suggests that there may be a group of bacterial species that reside or enter the oesophageal diverticulum like that of *C. capitata* and *B. oleae* [21, 22]. In the case of studies that were carried out on *C. capitata*, the most dominant bacterial species found in the oesophageal diverticulum were *Klebsiella oxytoca* and *Pantoea agglomerans* [21]*.* In the case of *B. oleae*, the species Ca. Erwinia dacicola, has been found to be the major symbiont of the fly and present in the oesophageal diverticulum [23–26, and]. This species is non-culturable whereas the two predominant bacterial species founds in *C. capitata* are. Structurally, however, they are all rod-shaped and between the size of 1-5 μm. The outcome of those studies indicate that any number of and any kind of bacterial species could be present in the oesophageal diverticulum of *D. pornia*. Culture dependent as well as culture independent studies, not reported here, were carried out to further identify, quantify as well as characterize the microbial community seen through the SEM studies on *D. pornia*.

The presence of yeast cells in the oesophageal diverticulum indicate that *D. pornia* either ingest them or they are inherited and that they may play some part in the digestive role of the fly. A recent study on yeast in *Bacterocera tryoni* (Froggatt) larvae suggests that a diverse group of yeasts are found in the alimentary tract [27]. The study was not clear on whether the yeasts found in the larvae were vertically transmitted, but it is suggested that the yeast is ingested by the larvae. It is evident from the diets that are provided to most laboratory reared Tephritids, that yeast play an important role in the nutrition of the fly. A further study on the role of yeast cells in *D. pornia* needs to be carried out as well.

An older study on the mating behaviour of *D. pornia* hypothesised that the nuptial gift provided by the male is produced in the salivary glands [16]. However, this is not proven with our new found understanding of the sexual dimorphism of the oesophageal diverticulum in adult *D. pornia*, we could add to the hypothesis that perhaps the oesophageal diverticulum of the male plays a role in providing/ supplying important bacteria for successful reproduction of the species. Two unique traits that *D. pornia* have that perhaps most other Tephritids do not, are a. sexual dimorphism with regard to the oesophageal diverticulum and b. the nuptial gift. We could speculate that these differences are connected to each other. A study to prove this hypothesis, however, needs to be carried out.

While our study has mainly focused on the location of the oesophageal diverticulum, its general structure and contents with respect to its relationship to symbiotic bacteria, we have not overlooked the potential importance of the crop. The crop has been studied in a few Tephritid species. It is believed to be a storage organ for food and bacteria [28–30]. The post-feeding bubble expelled by the adult flies contains bacteria that may be important for reproduction [29]. The importance and association of bacteria in the crop of adult *D. pornia* is yet to be studied.

Finally, the primary aim of these studies is to understand the relationship of bacteria with Tephritids and manipulate their symbiosis for the improvement of the current SIT. Recent studies have been made for the improvement of the SIT in *B. oleae, Glossina sp* (Diptera: Glossinidae)*, C. capitata* and *Bactrocera cucurbitae* [2, 3, 31–34]. In *C. capitata* and *B. cucurbitae* the symbiotic bacteria were provided as supplementary diets for the larvae and found that they contribute towards producing healthier sterile adults [32–34]. Similar studies need to be carried out on *B. tryoni.* With the help of current and future studies carried out on *D. pornia* we may be able to find symbionts that will ultimately help improve the SIT in *B. tryoni*.

## Conclusion

*D. pornia* has a digestive tract similar to that of other Tephritid species such as *C. capitata.* The oesophageal diverticulum of the adult *D. pornia* is located in the anterior area of the head, proximal to the left hemisphere of the brain and can be classified as ‘*Ceratitis’* type.

Micro-CT scans of soft tissues of Tephritid species can be conducted with a SkyScan 1072. The Avizo® Fire software can be successfully used to reconstruct 3D images of the CT scans of soft tissues of Tephritid species. Due to the ability to visualise CT images in 3D format, it allows for a better understanding of the overall morphology of organs such as the oesophageal diverticulum in a sensitive and complex structure. The methods used here have provided a guideline for future CT-based studies of all other Tephritid species.

The oesophageal diverticulum of *D. pornia* hosts a group of morphologically homogeneous rod-shaped bacterial cells and possibly some yeast cells. Recently developed molecular methods of bacterial identification and characterization may provide a greater understanding of the diversity of bacterial populations found within the oesophageal diverticulum of *D. pornia* as the next step in understanding the relationship between these bacteria and the host species. Further studies on the exploitation of the relationship between gut bacteria and fruit flies need to be carried out to successfully incorporate in the improvement of SIT and other fruit fly control techniques.

## Data Availability

The data files of SEM and micro-CT scans used in the current study are available from the corresponding authors on reasonable request.

## Abbreviations

3D: Three dimensional
ROI: Region of interest
SIT: Sterile insect technique
TIFF: Tagged image file format
